# Secondary Bilateral Cleft Lip Deformity Correction With Modified Double Z-Plasty to Reconstruct Cupid’s Bow

**DOI:** 10.7759/cureus.85267

**Published:** 2025-06-03

**Authors:** Hideto Imura, Le Kha Anh, Teruyuki Niimi, Tran Phuong Thao, Nagato Natsume

**Affiliations:** 1 Division of Research and Treatment for Oral and Maxillofacial Congenital Anomalies, School of Dentistry, Aichi Gakuin University, Nagoya, JPN; 2 School of Dentistry, Hanoi Medical University, Hanoi, VNM

**Keywords:** bilateral cleft lip palate, cupid's bow reconstruction, modified double z-plasty, secondary cleft lip palate surgery, technical report

## Abstract

A secondary bilateral cleft lip deformity represents a common aesthetic and functional complication following cleft lip surgery. Treatment approaches vary based on the severity of the deformity, with the surgeon determining the most appropriate intervention plan to address each case. Upper lip reconstruction following cheiloplasty faces significant challenges due to soft tissue deficiencies, scar formation, and unpredictable outcomes. This report introduces a novel method for minimally invasive upper lip reconstruction with bilateral cleft lip after initial cleft closure. This approach offers favorable aesthetic outcomes, a short operation time, a simple flap design, and minimal invasive intervention. Moreover, it can be combined with other procedures, such as scar removal on the white lip, correction of the red lip margin, oral vestibule formation, closure of the palatal fistula, and nasal correction, depending on the patient's situation.

## Introduction

Cleft lip and/or palate (CL/P) is a congenital malformation that represents a significant public health challenge [1]. This condition affects the physical appearance of individuals and has profound implications for their feeding, speech, hearing, and psychological well-being [2,3].

In particular, reconstructive lip anatomy in bilateral cleft lip patients is more challenging than unilateral cleft lip because of the soft tissue deficiency and the complexity of reconstructing lip morphology [4,5]. Secondary bilateral cleft deformities may be characterized by vermillion border deficiency, lip asymmetries, and expanded philtrum caused by the bidirectional pull of the orbicularis oris muscle [6]. Reconstructing Cupid's bow in patients with a bilateral cleft lip with or without a palate is crucial to achieving optimal aesthetics and functions. Several surgical techniques, such as Abbe Flap and Mulliken Method, can be employed to reconstruct Cupid's bow, focusing on symmetry, natural appearance, and optimal functionality. However, these methods still have some disadvantages, such as a two-stage procedure with Abbe Flap or difficulty in achieving Cupid's bow at the primary repair with the Mulliken Method [7,8]. We have developed and implemented a new method to improve aesthetic results called the modified double Z-plasty technique. This method features a simplified, minimally invasive design, allows for a swift surgical procedure, ensures patient comfort, and achieves vermillion and Cupid's bow symmetry.

In the present case, a new method for correcting important anatomical landmarks of the upper lip, especially Cupid's bow, in patients with a bilateral cleft lip is presented after three years of follow-up.

## Technical report

A female patient, diagnosed with bilateral cleft lip and palate (BCLP) at one month of age at Aichi Gakuin University Hospital, Nagoya, Japan, in 2015, underwent presurgical orthopedic treatment using a nasoalveolar molding (NAM) appliance to align the nasal cartilages and approximate the alveolar segments in preparation for cheiloplasty. Primary cleft lip repair was performed in 2016. Postoperatively, the patient exhibited residual aesthetic concerns, including nasal flattening, poorly defined upper lip contours, and distortion of key anatomical landmarks such as Cupid’s bow. At age five, in 2020, secondary lip revision was carried out using a modified double Z-plasty technique to improve upper lip morphology and achieve more favorable aesthetic outcomes.

**Figure 1:**
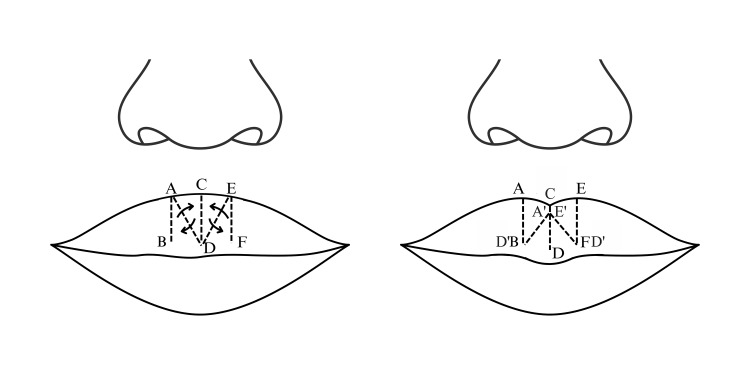
Modified double Z-plasty flap design Figure credit: Dr. Tran Phuong Thao

The procedure was performed under general anesthesia. First, a surgical skin marker pen was used to mark crucial bilateral anatomical landmarks to ensure symmetry, determine the surgical plan, and design the flaps. The Cupid’s bow peak was determined based on the white lip incision line, and the vermilion thickness guided the design of the Z-plasty incisions. Three equal and parallel Z-incision lines were drawn: two vertical lines flanking the Cupid’s bow peak and one midline incision forming the basis for the flap design (Figure 1).

**Figure 2:**
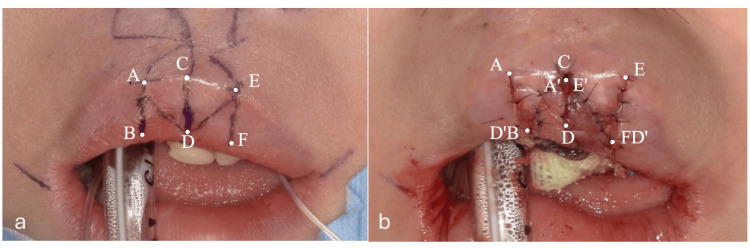
Double Z-plasty flap design and postoperative images a: flap design, b: post-operation

Local infiltration with 2% lidocaine containing 1:100,000 epinephrine (Lignospan Special, Septodont, France) was injected into the upper lip, followed by a gentle massage to promote diffusion and restore the natural tissue contour. Incisions were made with a No. 15 blade under the mucosa along the design line, including part of the muscle layer. After dissecting the surrounding tissue, the BAD and ADC flaps, as well as the CDE and DEF flaps, were cross-transposed and sutured at the muscle layer (Figure 2). The C point (central point of Cupid’s bow) was downward. Final closure was achieved with mucosal sutures using a 7-0 nylon suture, and an antibiotic dressing was finally applied to promote healing.

**Figure 3:**
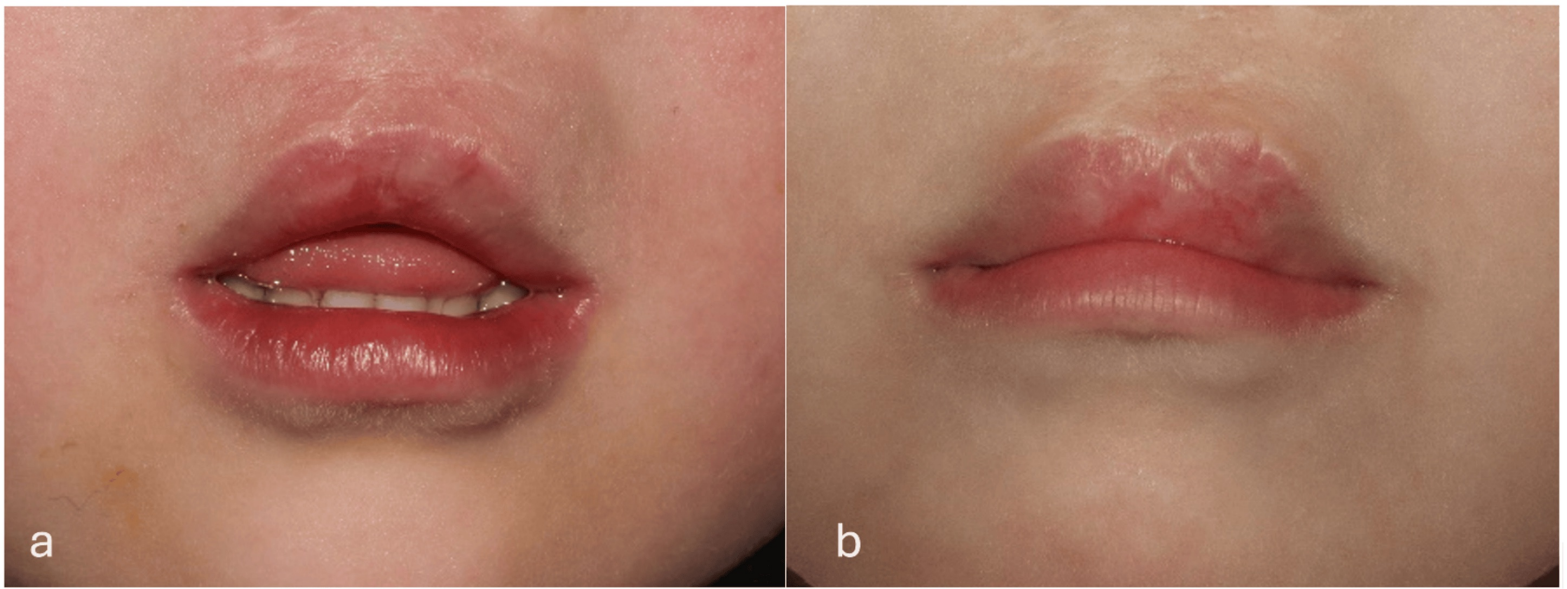
Images of the patient at pre-operation and the three-year follow-up a: pre-operation, b: Three-year follow-up

This technique advanced the central vermilion, enhanced the Cupid’s bow definition, and increased the prominence of the upper lip tubercle (Figure 3).

## Discussion

Cleft formation is influenced by the disruption of critical genetic pathways involved in craniofacial development. The transcription factor IRF6 (interferon regulatory factor 6) is a key regulator of periderm differentiation and epithelial adhesion [9]. Additionally, TFAP2A (transcription factor AP-2 alpha), which is critical for neural crest cell specification and migration, has been shown to regulate several downstream genes involved in craniofacial patterning [10]. Moreover, MEOX2 was identified as a potential gene for isolated cleft palate formation [11].

Although primary cleft lip repair aims for optimal outcomes, varying preoperative conditions may necessitate additional procedures. Postoperative complications, such as whistle deformity or incomplete Cupid’s bow formation, often due to soft tissue deficiency, can compromise facial aesthetics and philtral symmetry, posing significant morphological challenges [12].

In bilateral cleft lip patients, the absence of the philtrum and Cupid's bow often results from the underdevelopment or absence of the orbicularis oris muscle, leading to poorly defined upper lip morphology. Despite surgical efforts, the postoperative shape of Cupid’s bow is influenced not only by technique but also by the natural arc of the vermilion border. Secondary reconstruction methods are frequently required to improve function and aesthetics. The Abbe flap, which transfers tissue from the lower to the upper lip via a pedicled flap, offers favorable aesthetic outcomes and functional restoration, though its drawbacks include invasiveness, procedural complexity, and patient discomfort [7].

A free composite graft for cleft lip deformities, particularly emphasizing that grafting only the red lip improves blood circulation in the recipient site and maintains mucosal properties [13]. For patients with tight red lips and minimal midline vermilion, a satisfactory lip pout can be achieved without the Abbe flap by using tension-free grafting, which promotes high graft survival. However, this approach is limited by its suitability for small defects, potential color mismatch, and the need for additional donor sites.

For minor lip augmentation, free composite grafting is often utilized, whereas double Z-plasty is commonly applied for reconstructing Cupid's bow. Z-plasty, a well-established technique in plastic surgery, involves the transposition of two triangular flaps to facilitate tissue rearrangement and relieve tension. In our approach, symmetrical Z-plasty is employed to harness its lengthening effect, particularly in the central segment of Cupid's bow. However, a successful application requires adequate tissue availability in the direction of planned extension, making it most suitable for cases with sufficient upper lip tissue following flap creation and undermining of adjacent areas. However, other scar removal methods, such as laser therapy, should be considered to improve the final results.

In cases with limited mucosal tissue, a muscle-mucosal flap, including approximately 2 mm of underlying muscle, is harvested to enhance vascular supply. This technique, confined to the red lip region, is minimally invasive, simple, and rapid to perform. It can be integrated with adjunctive procedures, such as white lip scar revision, red lip margin correction, vestibular deepening, palatal pit closure, and nasal correction, depending on the individual clinical requirements.

## Conclusions

This method stands out from other surgical techniques due to its minimally invasive nature, ease of design, and short operation time. It can be performed independently or combined with other procedures such as rhinoplasty. The technique ensures good blood circulation to the flap, with no signs of partial mucosal necrosis post-surgery. This approach is highly effective, especially in cases where the upper lip lacks tightness. The three-year follow-up period showed the durability of Cupid's bow and the vermilion shape. However, the remaining scar is still challenging for optimal aesthetic results.
